# Quantitative detection of corrosion minerals in carbon steel using shortwave infrared hyperspectral imaging

**DOI:** 10.1039/d2ra05267a

**Published:** 2022-11-15

**Authors:** Thomas De Kerf, Arthur Gestels, Koen Janssens, Paul Scheunders, Gunther Steenackers, Steve Vanlanduit

**Affiliations:** Faculty of Applied Engineering, Department Electromechanics, Research Group InViLab, University of Antwerp Groenenborgerlaan 171 2020 Antwerp Belgium thomas.dekerf@uantwerpen.be; Department of Physics, AXIS Research Group, University of Antwerp Groenenborgerlaan 171 2020 Antwerp Belgium; Department of Physics, Imec-Vision Lab Research Group, University of Antwerp Edegemsesteenweg 200-240 2610 Antwerp Belgium

## Abstract

This study presents a novel method for the detection and quantification of atmospheric corrosion products on carbon steel. Using hyperspectral imaging (HSI) in the short-wave infrared range (SWIR) (900–1700 nm), we are able to identify the most common corrosion minerals such as: α-FeO(OH) (goethite), γ-FeO(OH) (lepidocrocite), and γ-Fe_2_O_3_ (maghemite). Six carbon steel samples were artificially corroded in a salt spray chamber, each sample with a different duration (between 1 h and 120 hours). These samples were analysed by scanning X-ray diffraction (XRD) and also using a SWIR HSI system. The XRD data is used as baseline data. A random forest regression algorithm is used for training on the combined XRD and HSI data set. Using the trained model, we can predict the abundance map based on the HSI images alone. Several image correlation metrics are used to assess the similarity between the original XRD images and the HSI images. The overall abundance is also calculated and compared for XRD and HSI images. The analysis results show that we are able to obtain visually similar images, with error rates ranging from 3.27 to 13.37%. This suggests that hyperspectral imaging could be a viable tool for the study of corrosion minerals.

## Abstract 2

This study presents a novel method for the detection and quantification of atmospheric corrosion products on carbon steel.

## Graphical abstract

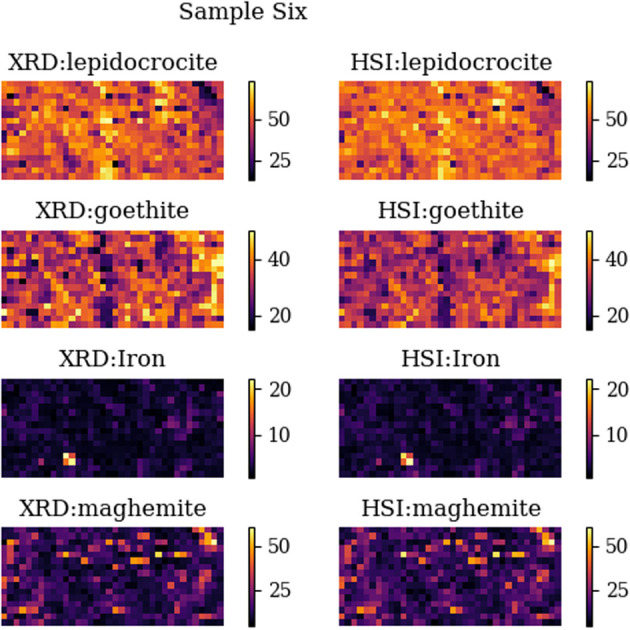

## 1 Introduction

**Fig. 1:**
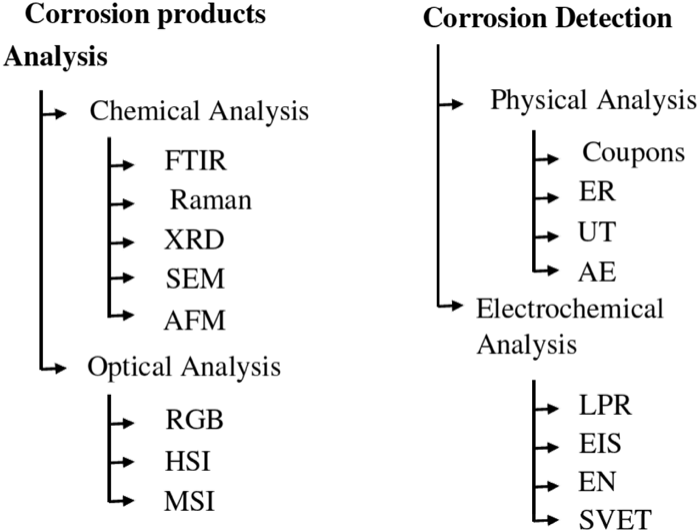
Overview of commonly used corrosion detection methods. Inspired by ref. 25.

Accurate and efficient detection of corrosion is critical to maintaining the health of metal structures and reducing the life-cycle cost of industrial infrastructure. The man-hours spent identifying the problem and then remediating it through a major overhaul or replacement of critical parts represent a significant portion of the lifecycle cost of all platforms and infrastructure.^1^ Therefore, early detection of corrosion problems reduces the total cost of ownership. There are several approaches to investigate atmospheric corrosion in carbon steel samples, which can be divided into two main categories: corrosion detection based on material changes and corrosion detection based on characterization of corrosion products. An overview of these techniques is given in Fig. 1.

Regarding the first category, the techniques can be divided into two subcategories: physical measurement techniques, which assess the damage caused by the corrosion, and electrochemical techniques, which assess the chemical change caused by the corrosion process. Proven physical measurement techniques include coupons, electrical resistance (ER),^2^ ultrasonic testing (UT)^3^ and acoustic emission (AE).^4^ These methods directly measure, in different ways, the damage caused by the corrosion process to the structure or sample under investigation. In general, these measurement methods can predict the corrosion rate in mm per year for a given location. This analysis can then be extrapolated to the entire structure. When using ER, AE and UT, the inspection of an entire structure is time-consuming due to its limited inspection area.

Electrochemical measurement methods include linear polarisation resistance (LPR), electrical impedance spectroscopy (EIS),^5^ electrochemical noise (EN)^6^ and scanning vibrating electrode technique (SVET).^7^ These methods measure the changes in the electrochemical signal in a material caused by the corrosion process. They are good for determining the overall corrosion rate of a structure, but they are not suitable for localising corrosion on a larger structure.

Another approach to corrosion detection is to identify, locate and quantify corrosion products. Correct identification and quantification of corrosion minerals has been correlated with corrosion rate in several research articles. A protective ability index (PAI)^8,9^ is proposed as a metric to evaluate the protective properties of a corrosion layer. Two categories can also be distinguished in the identification of minerals: chemical analysis on the one hand and optical imaging on the other. Common methods of chemical analysis include Fourier transform infrared spectroscopy (FTIR),^10^ Raman spectroscopy,^11^ X-ray diffraction (XRD),^12^ scanning electron microscopy (SEM),^13^ and atomic force microscopy (AFM).^14^ Previous studies have shown that each of these methods is able to identify the different corrosion minerals that form during atmospheric corrosion, such as α-FeO(OH) (goethite), γ-FeO(OH) (lepidocrocite), γ-Fe_2_O_3_ (maghemite). These are very accurate methods that are well suited to a laboratory environment with small samples. However, applying these techniques outside the laboratory would again be very time-consuming and practically unattainable.

Optical measurement techniques, on the other hand, can use larger sensor arrays to measure or image larger areas. Examples include standard visual spectrum (RGB) cameras, multispectral cameras (MSI) and hyperspectral cameras (HSI). Using RGB cameras to inspect corrosion minerals is a difficult task because the specific minerals all have the same red-brown hue.^15,16^

A compromise between optical imaging and chemical detection can be found in hyperspectral and multispectral imaging. With these cameras, we obtain more spectral information (typically 3–10 wavelengths for multispectral and more than 10 wavelengths for hyperspectral images) than using an RGB camera. These cameras can operate in different spectra, ranging from ultraviolet to long-wave infrared. Hyperspectral imaging is used in a variety of applications: remote sensing, food analysis,^17,18^ biomedical research, waste separation, agriculture.^19,20^ As for the detection of corrosion with HSI, research is limited. Halford *et al.*^21^ used a HSI, to study corrosion on bronze statues. Antony *et al.*^22^ used a HSI with a fibre bundle probe attached to detect corrosion on steel samples. Al Ktash *et al.*^23^ proposed a solution using UV HSI to characterise oxide layers on copper.

Previous research by the same authors.^24^ focused on the comparison between FTIR analysis and HSI measurements. However, the FTIR data were obtained with scraped corrosion products, so spatial information about where the corrosion products are present was not considered. This article fills this gap. We not only obtain the mineral amounts of the individual corrosion products, but also collect spatial information that can be correlated with the HSI measurements.

## 2 Materials and methods

### 2.1 Sample preparation

**Fig. 2:**
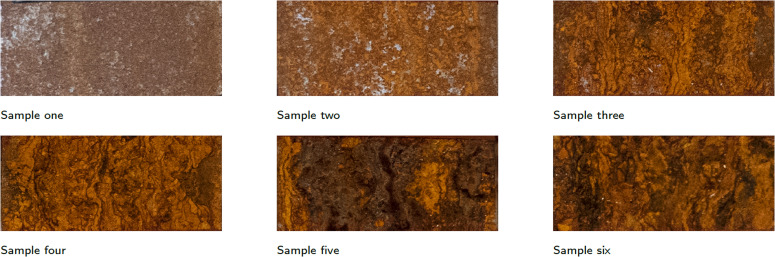
RGB images of the artificially corroded samples.

Cold rolled carbon steel samples with the following dimensions: 150 × 50 × 1 mm (length × width × thickness). The specimens were of DC01 quality as described in DIN EN 10130: 2006. The surface was then sanded with 400 and then 800 grit sandpaper. After sanding, the parts were first rinsed with demineralised water and then with an isopropyl alcohol cleaning solution. The samples were placed in the salt spray chamber at the same time. The accelerated corrosion test was conducted according to DIN ISO 9227,^26^ with a chamber temperature of 35 °C and a spray temperature of 45 °C. The pH was measured at one-day intervals and remained between 6.8 and 7.1. Each sample was taken out of the chamber at a different time, with a total salt spray duration of one hour for sample one and 120 hours for sample six. The durations of accelerated corrosion for all samples are given in Table 1. Fig. 2 shows the different samples with a clear difference in the degree of corrosion.

**Table 1:** Duration (in hours) of the accelerated salt spray test for each sample

Sample #	1	2	3	4	5	6
Duration (h)	1	2	24	48	72	120

### 2.2 XRD measurements

**Fig. 3:**
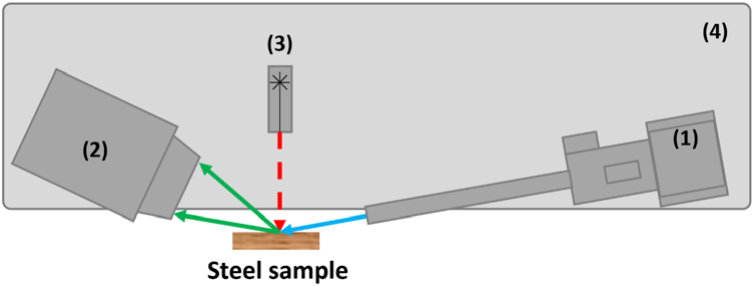
Schematic overview of the scanning XRD setup. With (1) as the X-ray source and optics, (2) XRD detector, (3) distance laser, and (4) the motorized platform.

**Fig. 4:**
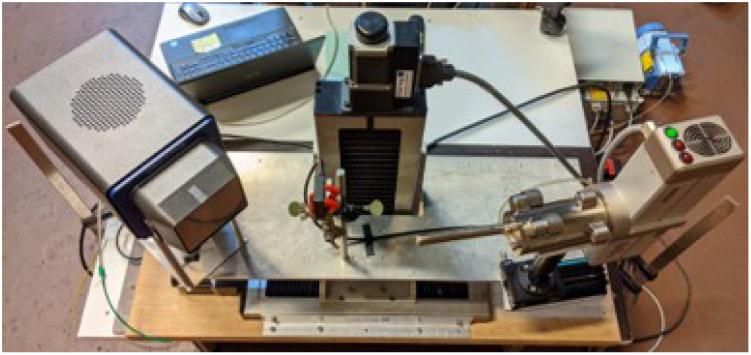
Image of the scanning XRD setup.

The MA-XRD measurements were performed by means of an in-house built mobile scanner (AXIS, University of Antwerp, Belgium). The device is set-up with a low power X-ray micro source (50 W, Iμ S–Cu, Incoatec GmbH, Germany), which produces a monochromatic and focused X-ray beam (Cu–Kα; 8.04 keV), a more detailed description can be found elsewhere^27^[24]. A primary beam angle of incidence of 10°relative to the corroded steel samples was employed due to geometrical limitations. This caused the beam footprint to become elongated in the horizontal direction so that it was of the order of 0.8 mm in the horizontal and 0.2 mm in the vertical direction. A PILATUS 200 K area detector was used to record 2D diffraction patterns for each irradiated position. To reduce the effect of local topography of the steel samples on the diffraction data, the distance between the artwork and the scanner was automatically adjusted with a laser distance sensor (Baumer GmbH, Germany) at each measurement point. All components are placed on a motorized platform that is capable of moving in the XYZ directions (30 × 30 × 10 cm^3^). The in-house developed software package XRDUA was used for the processing of all XRD data. XRDUA provides the necessary tools for extracting crystalline-specific distributions from the large number of 2D diffraction patterns obtained during XRPD imaging experiments.^28^ A schematic overview of the setup can be found in Fig. 3 and the actual setup in Fig. 4. A spatial resolution of 1.3 mm per pixel is obtained for this setup.

An example of the resulting XRD measurements can be found in the left column of Fig. 9.

### 2.3 HSI measurements

**Fig. 5:**
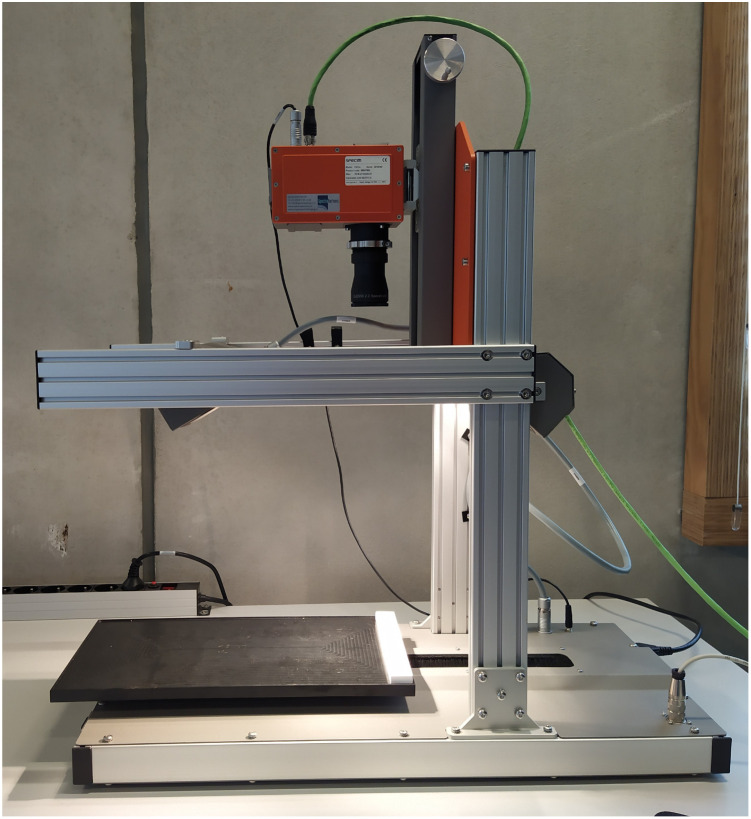
SPECIM FX17 and SPECIM Lab Scanner laboratory setup.

The hyperspectral images were acquired using a push-broom short-wave hyperspectral imaging system. An image of the setup can be seen in Fig. 5. The setup consists of a motorised translation stage (SPECIM laboratory scanner) that moves the sample while the camera is held in place. During the scanning process, the samples are illuminated with 6 halogen lamps with a power of 25 W. The camera (SPECIM FX17) captures 224 bands for each of the 640 pixels in the range of 900–1700 nm. The lens used for the measurements has a FOV of 12 °and an aperture of F/1.7. The distance between the lens and the samples was 200 mm, resulting in a spatial resolution of 0.17 mm per pixel. No spectral binning was applied, giving us a FWHM (full width at half maximum) of 8 nm. To convert the measured values into a reflectance value, a calibration was performed with a dark and a white reference. The dark reference was obtained by closing the shutter and averaging over 100 captured images. For the white reference, a Spectralon tile with a reflectance of 99% was taken and also averaged over 100 images. The scanned image was then corrected for each wavelength using the eqn (1).^29^1
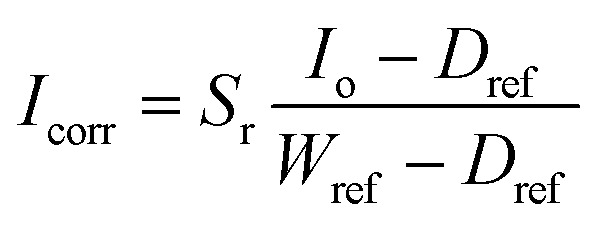
with *I*_corr_ as the corrected image, *S*_r_ as the reflectivity of the spectralon tile, *I*_o_ the uncalibrated image, *D*_ref_ as the averaged dark reference and *W*_ref_ as the white reference. The simultaneous control of the translation stage and the camera acquisition was done using Specim Labscanner software. A camera frame rate of 50 Hz and a scanning speed of 6.26 mm s^−1^ were used to scan all the samples. It required approximately 25 seconds to scan the entire sample. The post-processing and white calibration was done in Python.

Several post-processing steps are applied to the calibrated hyperspectral images. Since the first and last bands have a high noise/data ratio due to lower quantum efficiency at these wavelengths, these bands (the first and last 5 bands) are removed from the spectra. A Savitsky–Golay filter is also applied to each spectrum to smooth the spectra and remove unwanted irregularities that could affect classification accuracy. In a final step, the spectra are normalised between 0 and 1 to remove scale differences that may affect the machine learning algorithms.

### 2.4 FTIR measurements

A Macroscopic reflectance FTIR (MA-rFTIR) is used to collect spectra for each sample. The device consists of a portable bruker alfpha FTIR spectrometer that is positioned on a xy translation stage. Thus making it possible to do a pointwise scanning of larger 2D surfaces. The spectral resolution is 4 cm^−1^ and the spectral range spans from 375–7500 4 cm^−1^. Detailed information on the construction and implementation of this Ma-rFTIR scanner can be found in.^30^ The spatial resolution is kept similar to that of the XRD thus interpolation of the spectra is not needed. The spectra are smoothened with a savitsky golay filter with a window size of 25 and polynomial fit of the third degree. The postprocessing of the spectra was done using the Spectragryph software.^31^

### 2.5 Data analysis

**Fig. 6:**
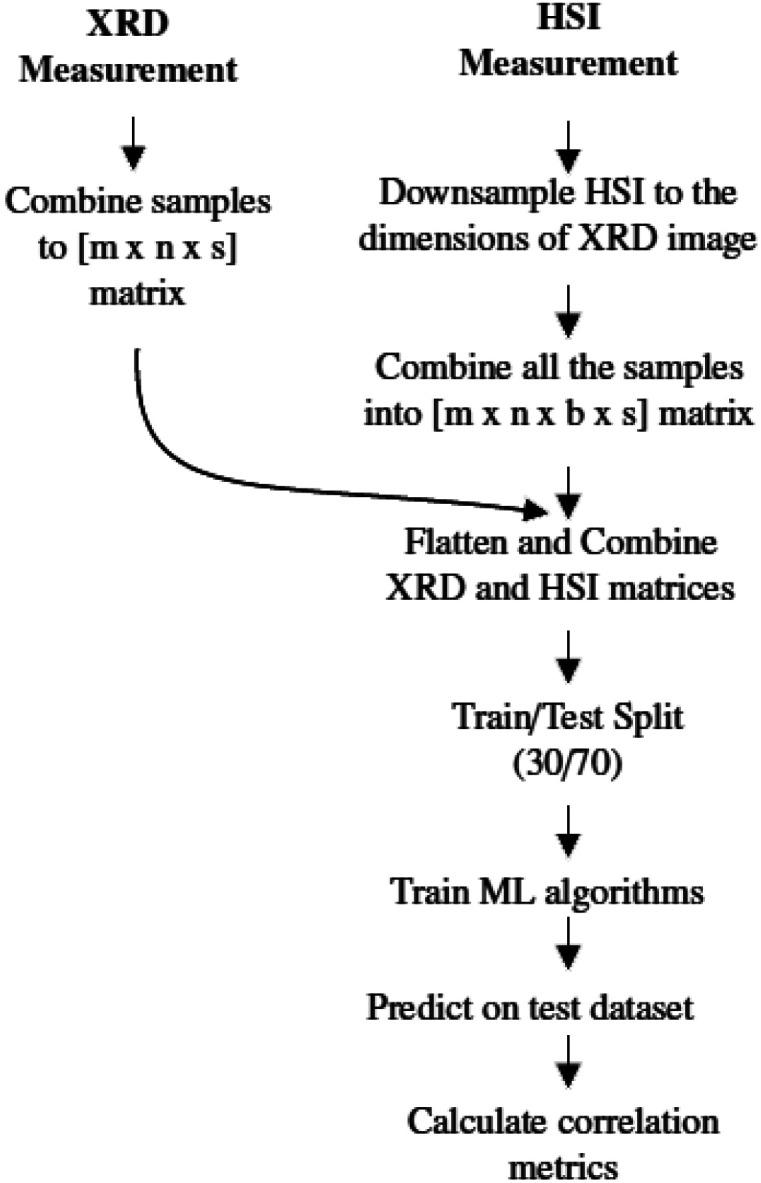
Flow chart of the steps to compare XRD and HSI measurement. This is done for each investigated mineral. With m and n as the horizontal and pixels dimensions, s as the number of samples and b the number of spectral bands.

The spatial resolution of the XRD measurements (1.3 mm per pixel) and the HSI (0.17 mm per pixel) measurements are not the same, so several steps must be taken to align these data. The methodology for aligning the two data sets is shown in Fig. 6 and the steps are explained in more detail in the next paragraph. It is important to note that these steps are repeated for each mineral, *e.g.* each mineral is processed and evaluated separately.

#### 2.5.1 XRD

For each sample, the mineral abundance data is loaded into a 2D matrix and used as the reference values. All samples are combined into a 3D matrix (*mxnxs*), where *m* and *n* are the number of pixels in *x*- and *y*-direction, respectively, and *s* is the number of samples.

The data from HSI is subjected to several preprocessing steps, such as calibration, removal of noisy areas and smoothing, as mentioned in Section 2.3. After these steps, the size of the HSI data cube is adjusted to the dimension of the XRD image. To reduce the size of the images, a bicubic interpolation method is used. The downsized data cubes are then combined into a 4D matrix (*mxnxsxb*), where *b* is the number of spectral bands. The next step is to combine and smooth the XRD and HSI data cubes. For both data cubes, the first three dimensions are transposed into a vector of size (*mxnxs*). In the end, we get a Y-vector containing the XRD data and a 2D matrix for the hyperspectral data. So for each hyperspectral pixel (spectrum) there is a corresponding abundance value that is measured by the XRD for that specific mineral. This flattening is necessary to use the machine learning algorithm. The resulting dataset is then split into a training and a testing part with a ratio of 30/70. A small training dataset is deliberately chosen to prevent overfitting the model.

A random forest regression^32^ is trained on these data. This algorithm uses several parallel decision trees whose results are averaged to achieve a higher overall prediction accuracy. A random subset of the dataset is used to create each decision tree. Averaging or ensemble methods are commonly used in machine learning to achieve higher accuracy compared to non-ensemble methods.^33^ As with most machine learning algorithms, there are a number of hyperparameters that can be defined and fine-tuned. A parameter optimisation algorithm ^34^ is used to determine the most successful set of hyperparameters for the model. Five-fold cross-validation is used to calculate the mean accuracy of the model.

After tuning the algorithm, we can use the best parameters to predict an abundance map based on the test dataset. The subset of the dataset on which the model was trained is not omitted when calculating the correlation metrics. The correlation metrics used are explained in more detail in the next section. All calculations were performed on a Windows 10 computer with an Intel Core i7-9750H with 12 cores and a speed of 2.6 GHz, 32 GB RAM and an Nvidia GTX 1650 graphics card. The open source Python package sci-kit learn^35^ was used to implement these algorithms.

### 2.6 Correlation metrics

Four different correlation metrics are used to evaluate the predicted HSI images with the XRD measurements. The use of the different correlation metrics is necessary because each metric evaluates similarity differently.^36^ The following metrics are used in this article: *R*^2^, RMSE, SSID and SRE. The first two can be classified as statistical measures, while the last two are more suitable for finding visual or feature correlations between images.

• The correlation coefficient or *R*^2^ will measure the correlation between the pixel values of the XRD image and the HSI image.

• The Root Mean Square Error (RMSE) is a measure for the difference in value on a pixel level between the two images. RMSE is an often used similarity comparison metric, however when the values are shifted slightly, this will have a major impact on the RMSE value. While the overall image will still have a similar appearance. RMSE is calculated using eqn (2)2
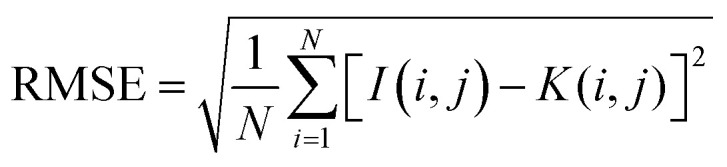
with *N* as the total number of pixels, *I* as the XRD image and *K* as the HSI image.

• Structural Similar Index Measure (SSIM)^37^ will focus on the perceptual difference between the images. This algorithm is traditionally used to assess the image quality after compression is applied. SSIM is calculated using eqn (3). The output is a value between −1 and 1 with 1 being a complete identical image.3
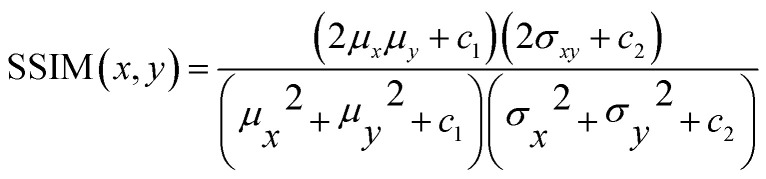
With *μ*_*x*_ the average of *x*, *μ*_*y*_ the average of *y*, *σ*_*x*_2 the variance of *x*, *σ*_*y*_2 the variance of *y*, *σ*_*xy*_ the covariance of *x* and *y*, *c*_1_ = (*k*_1_*L*)^2^, *c*_2_ = (*k*_2_*L*)^2^ two variables to stabilize the division with weak denominator, *L* the dynamic range of the pixel-values (typically this is 2^#bits per pixel^ − 1, *k*_1_ = 0.01 and *k*_2_ = 0.03 by default.

• Signal to Reconstruction Error ratio (SRE)^38^ measures the error relative to the power of the signal. The authors show that using SRE is better suited to make errors comparable between images of varying brightness. SRE is expressed in decibels (dB) and calculated with eqn (4)4
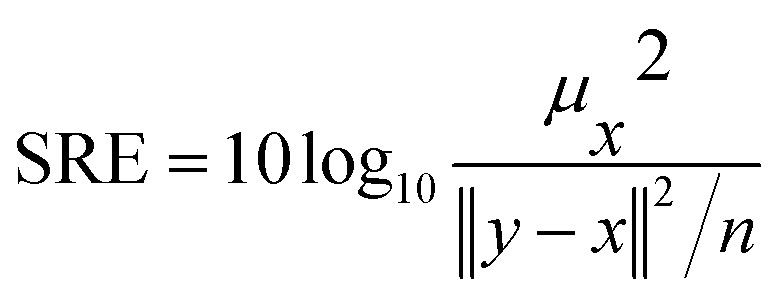
With *x* being the XRD abundance map, *y* the predicted HSI abundance map, *μ*_*x*_ the average value of *x* and *n* the amount of pixels in the XRD or HSI abundance map.

## 3 Results

### 3.1 FTIR analysis

**Fig. 7:**
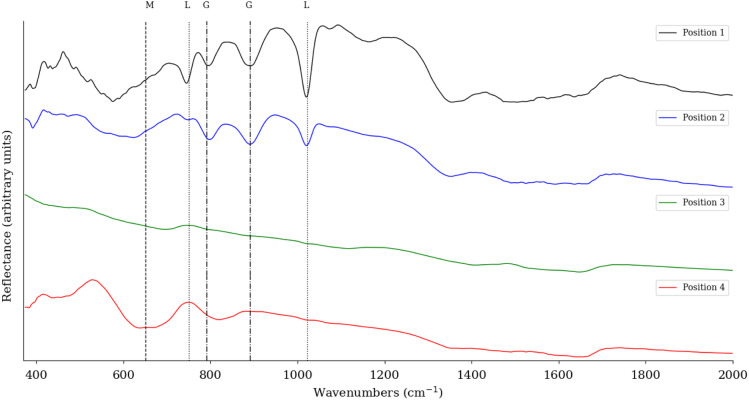
FTIR Reflectance spectra for four different positions in sample six. The vertical lines represent the distinct features that are described in the literature for each mineral. (G = goethite, M = maghemite, L = lepidocrocite).

**Fig. 8:**
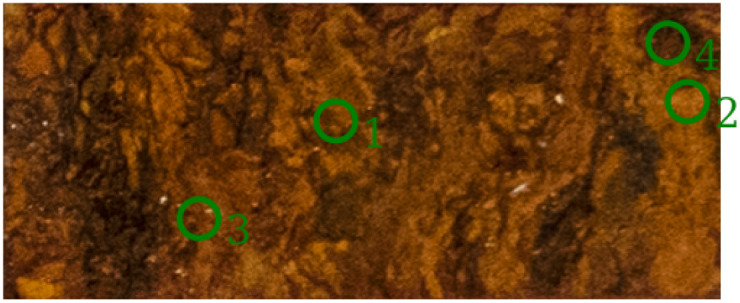
RGB image of sample six. The green circles indicate the FTIR measurement location.

To confirm the XRD analysis, FTIR is used to evaluate the minerals present in the sample. Sample six was measured with an FTIR scanner to obtain both spatial and spectral information. Fig. 7 shows the spectra from four different locations. These locations are indicated by circles on the RGB image in Fig. 8. The XRD analysis showed that the highest concentration for each of the categories was measured at these four locations. The different spectra show that when the abundance of a particular mineral is high, the distinct spectral features are also found in the FTIR spectra. The XRD measurements for position 1 show an abundance of 73.44% of lepidocrocite, which is confirmed by the pronounced spectral features at 1023 and 750 cm^−1^. Also present at this position is 22.54% goethite, which is also visible in the spectrum, with peaks in the range of 790 and 900 cm^−1^. For position 2, the XRD measurements indicate the presence of 54.22% goethite and 24.43% lepidocrocite. Looking at the FTIR spectra, we see that the spectra of both goethite and lepidocrocite are present. Compared to position 1, the features of goethite are more pronounced, indicating that a higher concentration of goethite is present. Position 3, as expected, has no significant features consistent with the corrosion spectra, being identified as iron in the XRD analysis. Position 4 has a broad feature in the 620–660 cm^−1^ range indicating the presence of maghemite. The XRD measurements show that maghemite is indeed the most abundant mineral at this position.

### 3.2 Visual comparison

**Fig. 9:**
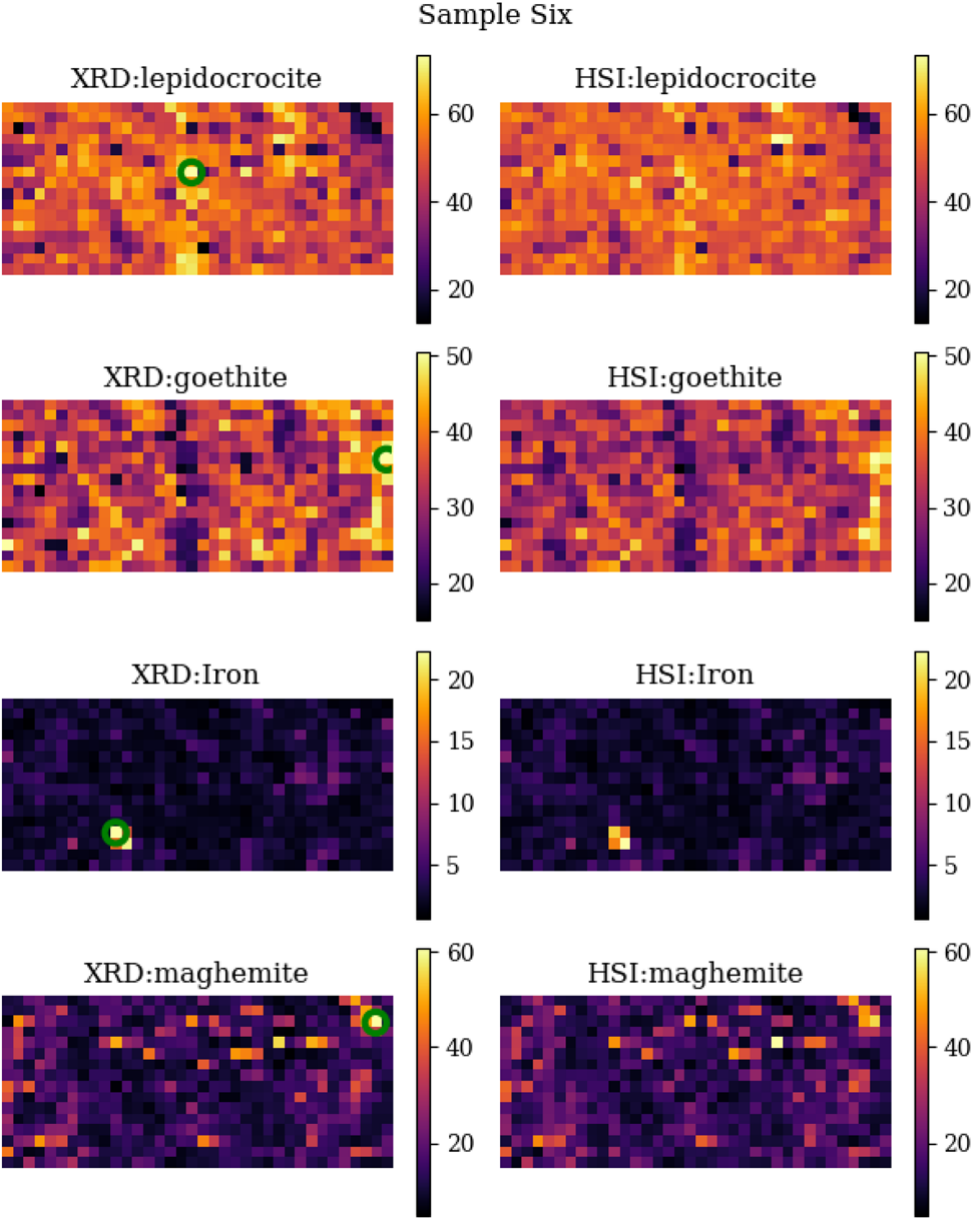
Side by side comparison of the individual XRD abundance maps and the predicted abundance maps calculated by the machine learning algorithm (random forest regressor) for sample six. The green circles represent the locations with the highest abundance of that specific mineral. The FTIR spectra of these locations are displayed in Fig. 7.

A comparison between the measured XRD abundance maps and the predicted abundance maps from the HSI measurements for sample six can be found in Fig. 9. From this comparison, we see that they look quite similar from a visual point of view. The overall intensity of the images is the same, and they also have the same features (light and dark spots). On closer inspection, we can see that there are some differences in the location of the above features. For example, the iron spot in the lower left area is clearly 4 pixels in size, whereas the prediction from HIS predicts only 2 pixels as a large iron concentration. This obviously has a very large impact on the comparison metrics and also on the overall error between XRD and HSI abundance area. It is clear from the comparison that the two most important corrosion minerals are lepidocrocite and goethite, and to a lesser concentration also maghemite, while the presence of iron is almost negligible.

### 3.3 Analytical comparison

The results of the HSI and XRD comparison are shown in Table 2. For sample one, only iron is detected from the XRD measurements. However, the RGB images in Fig. 2a show that there is corrosion present, but this is very early stage corrosion (after one hour). The signals of iron are abundantly in the resulting diffractogram, thus making it impossible to distinguish this early stage corrosion from the background using XRD. Therefore, sample one is not included in the overview table. For each sample and mineral, the *R*^2^, RMSE, SSIM and SRE values were calculated. When looking at the correlation value of *R*^2^ in Table 2, it is noticeable that there are large differences between the different minerals and samples. In particular, the *R*^2^ values for iron are remarkably low. This can be explained by the small amount of iron abundance data available for this sample. Another possible to source of error is that iron is difficult to predict because of the difference in sampling depth between the different methods. Iron will be the layer that at the deepest probing depth. Thus, its abundance depends on the corrosion layers on top.

**Table 2:** Results of the predicted hyperspectral abundance maps, evaluated using four different correlation metrics. The values marked in bold show anomalous values and indicate a bad performance of the regression analysis. In the last column, negative percentages indicate an underprediction of the total abundance, whereas positive values demonstrate an overprediction

		Random forest regression				Total abundance	
		*R* ^2^	RMSE	SRE	SSIM	XRD abundance (%)	Difference XRD *vs.* HSI (%)
Sample one	Iron	0.74	0.07	43.68	0.81	100	−25.23
	Lepidocrocite	—	—	—	—	—	—
	Goethite	—	—	—	—	—	—
	Maghemite	—	—	—	—	—	—
Sample two	Iron	0.54	0.05	44.95	0.83	66.26	1.97
	Lepidocrocite	0.62	0.07	41.18	0.83	33.74	−1.25
	Goethite	—	—	—	—	—	—
	Maghemite	—	—	—	—	—	—
Sample three	Iron	**0.13**	0.10	31.72	0.73	12.32	−3.27
	Lepidocrocite	0.55	0.06	44.31	0.83	40.67	4.79
	Goethite	0.62	0.08	44.19	0.87	31.45	1.21
	Maghemite	0.57	0.05	40.41	0.82	15.56	**−10.80**
Sample four	Iron	**−1.1**	0.14	26.45	0.73	5.21	−3.85
	Lepidocrocite	0.41	0.09	44.91	0.80	49.26	−0.19
	Goethite	0.53	0.08	43.20	0.83	32.43	0.40
	Maghemite	0.36	0.09	31.85	0.73	13.10	3.28
Sample five	Iron	**0.29**	0.09	**24.30**	0.87	2.39	**13.37**
	Lepidocrocite	0.67	0.06	39.92	0.88	31.59	−2.70
	Goethite	0.70	0.08	42.20	0.89	35.73	0.19
	Maghemite	0.67	0.05	39.97	0.89	30.28	1.72
Sample six	Iron	0.59	0.08	32.05	0.89	2.35	**13.86**
	Lepidocrocite	0.69	0.06	45.04	0.91	32.82	−0.63
	Goethite	0.72	0.05	42.28	0.88	34.80	−1.25
	Maghemite	0.69	0.07	35.59	0.89	30.03	5.41

**Fig. 10:**
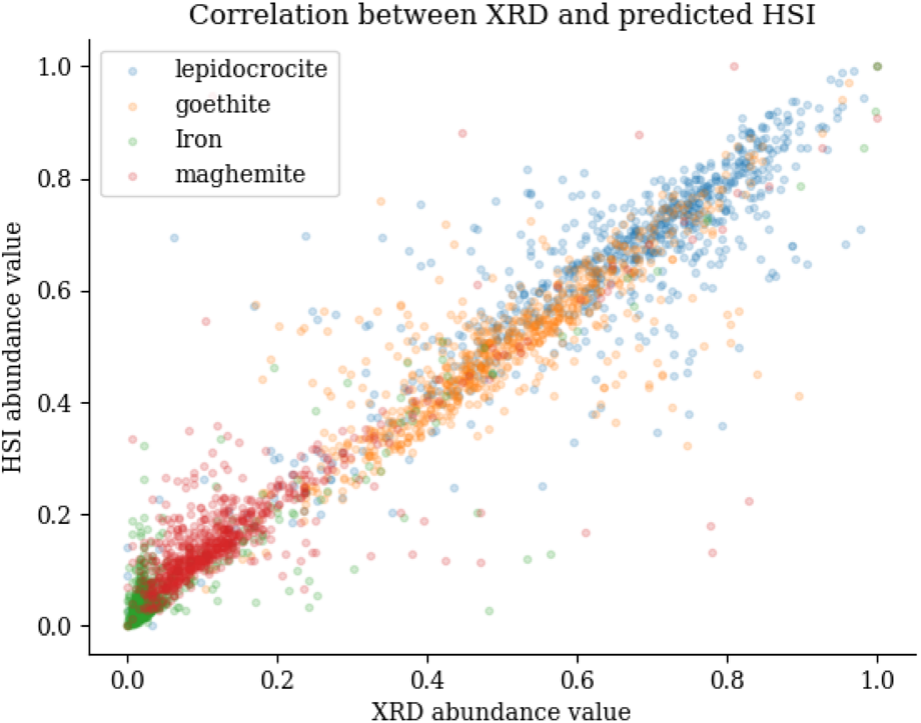
Correlation plot between abundance values of the XRD measurements and the predicted HSI measurements.

This can be seen in Fig. 10, which shows the abundance values for the XRD (*y*-axis) and HSI (*x*-axis). It is noticeably that the iron category is mainly located in the lower left corner and is quite distributed, which has a large impact on the *R*^2^ metric. When calculating the RMSE metric, the normalised values between 0 and 1 are used. This explains the overall small values. The largest values occur in the iron abundance maps, this is due to the limited variance of the data points for iron. This metric does not penalise individual clusters that are misclassified, as these rare large errors are offset by frequent small errors. This highlights the disadvantage of using the RMSE for this particular type of correlation metric. When looking at SRE scores, the differences are greater compared to the RMSE. SRE corrects for a shift in intensity so that the mediation effect of larger scores is offset. The SRE scores show that iron is again difficult to predict, as evidenced by the fact that it consistently scores the lowest. When comparing the three corrosion minerals, maghemite has the lowest, though still acceptable, score. Finally, the SSIM metric shows that the differences are very small when looking at the main features in the picture. This conclusion can also be drawn by looking at the comparison image in Fig. 9, where the main features are the same between the XRD and HSI images.

### 3.4 Total abundance comparison

Next to the visual comparison of the predictions *versus* the ground truth XRD values, it is also possible to express the abundance per mineral as a percentage of the total abundance. This value shows how much of the mineral in a sample is identified through XRD. We can compare these total abundance values from the XRD measurements to the total abundance values of the HSI predicted images and subsequently calculate the percentage error between them. These values are displayed in the last two columns of Table 2.

As can be seen from the table, the amount of iron steadily decreases, while the total abundance of the corrosion minerals increase. The order in which these minerals form (lepidocrocite → goethite → maghemite) has been previously shown in the literature, and this order is also apparent from the XRD and HSI measurements. As previously mentioned, the XRD measurements for sample one only contained iron. No other mineral were to be found. Very early stage corrosion was not detected using XRD, this explains the very large error between XRD and HSI.

What stands out is that if the XRD abundance is large, the difference between XRD and HSI will be smaller. The only exception is the maghemite in sample three with an XRD-HSI difference of 10.8% while still having an overall XRD abundance of 15.56%. A possible explanation of the large errors for iron and maghemite is that these categories show no distinct spectral features in the SWIR range. This makes it very difficult to distinguish between the two categories. When the total XRD abundance is larger than 20% we see that the overall differences between XRD and HSI are small, ranging from −2.7 to 5.41%. These low error figures, show that HSI can be an accurate replacement to detect iron compounds in the corrosion samples.

## 4 Conclusions

In this study, we assessed the use of hyperspectral imaging for the quantification of corrosion minerals in carbon steel samples. Using ground truth from scanning XRD measurements, we are able to create a machine learning model based on the hyperspectral measurements. The results show that this model is able to achieve a good correlation between the predicted hyperspectral abundance maps and the XRD measurements, for each of the corrosion minerals. The largest discrepancy between XRD and HSI is the fact that XRD is not able to identify very early stage corrosion. In sample one, after one hour of continuous salt spray, we visually see that there is a large corrosion patch, and this is also apparent in the hyperspectral predictions.

This study was limited by the absence of long stage, multilayered atmospherical corrosion. When the corrosion process is more matured, the literature shows that a multilayered corrosion structure occurs. This multilayered corrosion structure was not accounted for in this study, and it is possible that the performance of the proposed hyperspectral imaging method will decrease when measuring subsurface corrosion mineral compositions. Notwithstanding these limitations, the study suggests that for early stage corrosion, hyperspectral imaging can be used to quantify early stage atmospherical corrosion on carbon steel. Considering the large scanning area for the hyperspectral camera, ease of use, and portability, it is a feasible option to use in a non-laboratory setting. More research could be done using more advanced artificial intelligence methods such as deep learning architectures, yet these methods typically require more data to provide a benefit over more traditional machine learning methods that have been implemented in this article.

## Author contributions

De Kerf Thomas: methodology, writing – original draft, software, visualization, investigation. Arthur Gestels: software, investigation, writing – review & editing. Gunther Steenackers: supervision, funding acquisition, resources. Koen Janssens: supervision, funding acquisition, writing – review & editing, resources. Paul Scheunders: conceptualization, supervision, funding acquisition, writing – review & editing, resources. Steve Vanlanduit: conceptualization, supervision, funding acquisition, writing – review & editing, project administration, resources.

## Conflicts of interest

There are no conflicts to declare.

## Supplementary Material
